# Evaluating the performance of optically pumped magnetometers (OPM) in the diagnosis and presurgical workup of focal epilepsy

**DOI:** 10.1007/s00415-024-12196-5

**Published:** 2024-02-12

**Authors:** Phil Sequeiros, Khalid Hamandi

**Affiliations:** 1 The Wales Epilepsy Unit, Department of Neurology, University Hospital of Wales, Cardiff, CF144XW Wales; 2 Cardiff University Brain Research Imaging Centre, Cardiff University, Maindy Road, Cardiff, CF244HQ Wales

## Introduction

Magnetoencephalography (MEG) is a brain imaging technique used for measuring the weak magnetic fields at the scalp surface caused by underlying neuronal activity. These electromagnetic fields derive from the mass activity of dendritic currents in similarly orientated pyramidal neurons in the cerebral cortex [similar to the electroencephalography (EEG) signal]. Around 50,000 active neurons are needed to generate a detectable signal, measured in femto-Tesal, several orders of magnitude smaller than background environmental magnetic noise, hence the need for a shielded room for MEG. A SQUID (superconducting quantum interference device) is a very sensitive magnetometer which is used to measure these weak magnetic fields. MEG advantages over EEG include improved source localisation, and less motion artefact.

SQUID–MEG sensors need to be submerged in liquid helium at a temperature of -269 °C to achieve superconductivity, and are arranged in a helmet (or Dewar) of more than 300 sensors. SQUID–MEG as part of epilepsy surgery evaluation, is standard in most centres in the USA, and is generally linked to research programmes elsewhere in the world, due to the cost and maintenance of MEG scanners.

Recently, optically pumped magnetometers (OPM) have been described and developed. ‘Optically pumped’ refers to the use of a laser to pump atoms into a specific quantum state. The magnetised atomic vapour interacts with an external magnetic field passing through the sensor that modulates the light passing through the vapour, that is measured at a photodiode, to infer the magnetic field and underlying neural activity changes. OPM–MEG sensors operate at room temperature, obviating the need for helium supercooling, the Dewar and large machines. OPM–MEG offers portability similar to EEG, and data acquisition is quicker and better tolerated, given the use of a helmet compared to the individual electrodes and ‘scalp prep’ needed for EEG. Sensors are embedded in a helmet which is easily placed on the head by an operator closer to the scalp surface, offering greater signal to noise ratios, is more resilient to head motion than conventional MEG, and OPM helmets can be tailored to head size and shape, a particular advantage in children.

Three papers are selected from the earliest reports of OPM in epilepsy. The first paper is a proof of concept case report, the second and third papers compare SQUID–MEG and OPM–MEG in small case series.

## Optically pumped magnetoencephalography in epilepsy

In 2020, Vivekananda et al. demonstrated the first use of OPM–MEG in a patient with medically refractory, focal epilepsy at University College London. At that time, the clinical use of OPM–MEG had only been demonstrated on rodent models. The single study participant was a 47-year-old female who had meningitis at the age of 18 months. This patient experienced 10 focal impaired-aware seizures a day, refractory to multiple medications. Previous MRI brain demonstrated right-sided parieto-occipital damage.

She then underwent 30-min recording sessions with OPM–MEG within a magnetically shielded room. One session used a 3D-printed scanner cast, designed with patient-specific anatomical measurements taken from a previous 3 T MRI scan, with 15 s-generation OPM sensors. She was able to move her head freely throughout the recordings. Nineteen epileptiform spikes were identified, and localised activity to the right posterior quadrant, concordant with MRI and previous EEG recordings. The patient went on to undergo resective surgery for this region, with improvement in her seizure frequency and severity.

*Comment* Vivekananda et al. successfully demonstrated for the first time in humans that OPM–MEG can be used to detect abnormal interictal activity with similar morphology and consistency as identified by EEG. The technology would benefit from direct comparison between OPM–MEG and SQUID–MEG, and in larger cohorts, related to clinical diagnoses and outcomes [1].

## Non-invasive measurements of ictal and interictal epileptiform activity using optically pumped magnetometers

In 2023, Hillebrand et al. evaluated the clinical performance of a 12-channel OPM system compared to a conventional 306-channel SQUID–MEG system within a larger population number of seven patients with drug-refractory epilepsy. These participants had already undergone evaluation with clinical SQUID-based MEG prior to the study, and interictal epileptic discharges (IEDs) had been identified.

The team designed and manufactured 3D-printed helmets on the basis of participant’s anatomical measurements, taken from MRI scans during clinical work-up. OPM recordings were performed for participants in the morning with participants in a seated position, with SQUID–MEG being performed later in the afternoon with participants in the supine position. IEDs were then visually identified by an EEG/MEG technician. For the OPM data, field maps of IEDs identified in the SQUID data were used as reference and the OPM data were projected onto the SQUID-sensor layout to identify true positive IEDs.

*Comment* This study demonstrated consistency between OPM data and data generated by the conventional SQUID–MEG system, both in time and space. The authors also praised the feasibility of the OPM system, with the individualised helmet design improving the quality of recordings in the participants that experienced hyperkinetic seizures. They demonstrated that in one patient, the OPM also allowed for accurate reconstruction of seizure propagation patterns.

One limitation that could have potentially impacted the reliability of SQUID–MEG performance is how recordings of each system were taken at different times in the day, and with different patient positionings. The authors noted that this may have contributed to participant drowsiness during SQUID recordings, increasing the yield of epileptiform abnormalities. Also, each participant had already undergone successful clinical MEG prior to the study, with some having also undergone invasive EEG monitoring. Therefore, IEDs had already been identified in the study participants, which allowed for strategic placement of the limited number of six of the OPM sensors in this early proof of concept study [2].

## On-scalp optically pumped magnetometers versus cryogenic magnetoencephalography for diagnostic evaluation of epilepsy in school-aged children

This prospective study focused on the use of OPM and SQUID–MEG in the paediatric setting. As previously discussed, SQUID–MEG needs cryogenic cooling. As such, it requires the existence of a thermally insulated gap between the patient’s scalp and each individual SQUID, meaning that the brain-to-sensor distance averages at 2–5 cm in adults, and even more so in children. This increased distance detrimentally impacts the strength of the magnetic field strength. One of the notable advantages of OPM over SQUID–MEG is its ability to be adapted to the size and shape of different patients, with each OPM sensor being situated directly onto the patient’s scalp. This feature is highly invaluable in a setting where technology needs to allow for a high degree of anatomical variability. The authors utilised a 3D printed head case that housed 32 OPM sensors for a relatively small study population of five children, with an age range of 5–11 years.

*Comment* Despite the smaller number of 32 OPM sensors against the 306 SQUID sensors, the authors demonstrated that OPM–MEG provided higher amplitudes of interictal epileptic discharges (2.3–4.6 times higher, *P* < 0.001) and higher IED signal-to-noise ratios with similar localisation values to SQUID MEG. The authors comment that this was likely due to the reduced brain-to-sensor distance afforded by using OPM sensors, an average of 29.4mm with OPM compared to 57.6 mm with SQUID. There was also increased signal interference and subsequent noise artefacts with the OPM sensors, as participants were allowed to move freely. This was avoided by SQUID–MEG, where sensors were fixed and subjected to denoising software. Authors commented that this disadvantage could be negated with future use of OPM with the use of similar denoising software, algorithms and active shielding [3].

## Summary

There is much excitement around OPM–MEG which is not limited to epilepsy, but also in the study of brain oscillations and activity across a number of disease areas; main examples being dementia, traumatic brain injury, autism and schizophrenia, as well as the study of normal brain function. The technology remains at an early stage. The papers presented here use 6–32 on-scalp sensors, but 64-sensor systems have since been built and are in use, along with advances in shielding and motion correction. The full potential for OPM–MEG as a ‘standard’ clinical tool is still developing, and will no doubt accelerate; hardware and software solutions are tractable and OPM may soon become another tool in any hospital’s neurophysiology department.

## Data Availability

Not applicable.
